# Sexting Profiles, Mental Health and Quality of Life in Adolescents and Emerging Adults

**DOI:** 10.1007/s10964-026-02360-7

**Published:** 2026-05-18

**Authors:** Mónica Ojeda, Daniela Villa-Henao, Esperanza Espino, Rosario Del Rey

**Affiliations:** 1 Department of Developmental and Educational Psychology, Faculty of Educational Sciences, Universidad de Sevilla, Seville, Spain; 2 Department of Developmental and Educational Psychology, Faculty of Psychology, Universidad de Sevilla, Seville, Spain; 3 Department of Psychology, Faculty of Psychology and Education, Universidad Loyola Andalucía, Seville, Spain; 4 Department of Developmental and Educational Psychology, Universidad de Sevilla, C/ Camilo José Cela, s/n. 41018, Seville, Spain

**Keywords:** Sexting, Adolescents, Emerging adults, Mental health, Sexual double standards

## Abstract

Although sexting has become increasingly normalized among youth, less is known about how distinct patterns of sexting involvement relate to mental health and quality of life across developmental stages, and how gendered sexual norms shape these associations. This study analyzed a nationally representative sample of 3,534 Spanish adolescents and emerging adults aged 14–25 (M = 19.8, SD = 3.1; 49% girls/women) who reported engaging in at least one sexting behavior; analyses were conducted separately for adolescents (14–19 years) and emerging adults (20–25 years). Latent profile analyses identified two profiles in each age group. An Occasional Primary Sexting profile showed low involvement in all sexting behaviors (sending, receiving, non-consensual forwarding, and victimization), mainly reflecting occasional, consensual exchanges of one’s own erotic content. In contrast, a Frequent Extensive Sexting profile showed high and generalized engagement across all behaviors, with especially frequent receiving of sexual content, including both directly sent and forwarded material, along with increased involvement in non-consensual forwarding. Frequent Extensive Sexting was consistently associated with poorer quality of life and higher depression, anxiety, and stress. Endorsement of sexual double standards mediated anxiety in adolescents, and depression, anxiety, and stress among emerging adults. Additional analyses revealed notable gender differences, with girls/women reporting poorer mental health and boys/men exhibiting higher endorsement of sexual double standards, with indirect effects through these standards being particularly evident among them. These findings highlight the importance of considering heterogeneous patterns of sexting involvement and the role of sexual double standards in understanding how digital sexual behaviors are linked to youth mental health and quality of life.

## Introduction

Sexting, defined as the exchange of erotic, sexually suggestive or explicit content via digital devices, has become increasingly common during adolescence and emerging adulthood, two developmental periods marked by heightened socio-sexual exploration and vulnerability (Klettke et al., 2019). Factors such as age and gender could also shape these practices. Prevalence increases markedly from early adolescence, where 21.5% report sexting to emerging adulthood, where rates exceed 70% reflecting a developmental progression in these practices (Resett, 2019). Moreover, girls and women appear to be disproportionately affected by coercive, and by other non-consensual forms of sexting (Gassó et al., 2020). Despite growing attention, important gaps remain: most research is descriptive, limited to specific populations, and rarely adopts person-centered approaches that capture distinct sexting profiles and their psychosocial impact. In addition, although cultural and gender norms are known to shape sexting practices, particularly the sexual double standards, understood here as the degree to which individuals personally endorse unequal norms that reward or penalize the same sexual behavior depending on gender, their role in amplifying psychological consequences has received little empirical attention. This study identifies sexting profiles, examines their associations with quality of life and mental health, and tests the mediating role of sexual double standards in a nationally representative sample of Spanish adolescents and emerging adults, including gender differences.

### Sexting as a Multidimensional Phenomenon

Over the past two decades, sexting has gained visibility in adolescent and emerging adult health research, becoming a subject of academic and clinical debate (Livingstone & Görzig, 2014). While initially conceptualized as a risk behavior linked to cyberbullying and online victimization, more recent approaches position sexting along a continuum ranging from consensual sexual exploration to coercive practices with mental health implications (Chatzinikolaou & Lievens, 2021).

Multiple studies indicate that sexting is a multidimensional behavior expressed through various forms of involvement, such as active (sending), passive (receiving), or coerced participation, meaning participation that occurs under pressure or without full voluntary consent (Alonso & Romero, 2019; Resett, 2019). These forms are driven by varied motivations that shift across developmental stages, including seduction, affective validation, entertainment, body image expression, peer pressure, and coercion (Alonso Ruido, 2017; Ayala et al., 2019). This heterogeneity has led several authors to examine sexting profiles. For example, studies using sending and receiving behaviors among emerging adults have identified four profiles: no sexters (71.5%), word-only sexters (14.5%), frequent sexters (8.5%), and hyper sexters (5.5%) (Galovan et al., 2018). Other research has also identified and described prevalences in active, passive, and reciprocal sexting among adolescents and university students (Gassó et al., 2020). Although these studies represent important first steps toward recognizing variability in sexting practices, differences in the behaviors analyzed, measurement, and analytic approaches limit comparability. As a result, key questions remain unresolved, such as how different sexting behaviors co-occur and whether similar profiles emerge across developmental stages.

At the same time, despite the rapid expansion of research on sexting, much of the existing work remains descriptive or based on predefined behaviors or categorical classifications rather than person-centered approaches. This limits the ability to identify differentiated behavioral subgroups and examine how they relate to psychosocial correlates and well-being (Gassó et al., 2020). This gap is particularly relevant given the high prevalence of sexting reported among adolescents and emerging adults. For example, in a study of adolescents aged from 12 to 18 years, 95.5% reported having engaged in sexting at some point (Giménez-Gualdo et al., 2022). Specifically, 40% reported feeling pressured to publish or share their own erotic or sexual messages or images, 32.9% had voluntarily sent such content to others, and 94.3% had received it. Among college students, comparable patterns emerged, with 72% having engaged in sexting, including 37.1% who sent and 60.3% who received explicit content (Gassó et al., 2020). Yet, few studies have simultaneously compared adolescents and emerging adults, incorporated comprehensive behavioral indicators, including non-consensual practices, or examined how distinct sexting profiles relate to quality of life and mental health, leaving significant conceptual and empirical gaps unaddressed.

### Gender, Sexual Double Standards, and Mental Health Outcomes

Gender differences in sexting patterns are also well documented. Overall, boys are more likely to engage proactively (by sending, requesting, or sharing content), whereas girls are more frequently subject to unsolicited content, pressure, coercion, and social consequences (Frøyland et al., 2024; Gassó et al., 2020). Recent studies further indicate that girls experience more negative emotional reactions to sexting, regardless of their level of consent or role (Lebedíková et al., 2025). These differences are particularly pronounced during adolescence, with sustained higher involvement among boys, and persist into emerging adulthood (Cujano Chimborazo & Valencia Cepeda, 2024; Gassó et al., 2020).

These disparities are strongly shaped by cultural and educational norms that differentially sanction sexual behavior by gender. Boys tend to view sexting as a means of validation or intimacy, while girls more often anticipate reputational harm, even when participation is voluntary (Alonso Ruido, 2017). This asymmetry reflects the enduring influence of the sexual double standards, a structural form of inequality where women’s sexual expression is penalized and men’s tolerated or rewarded (Gassó et al., 2020). This dynamic not only influences perceptions of risk but may amplify the psychological consequences of sexting, particularly among girls and young women, increasing feelings of shame, anxiety, and social stigma (Ayala et al., 2019). However, to date, no studies have empirically examined the mediating role of the sexual double standards in the link between sexting and quality of life and mental health.

The association between sexting and mental health has garnered growing attention, with research showing that adolescents who engage in sexting, particularly in non-consensual behaviors or alongside other risk behaviors, report higher levels of depressive symptoms and other psychosocial difficulties (Frankel et al., 2018; Frøyland et al., 2024). These effects tend to be more pronounced among girls and young women, who report greater emotional distress, shame, and reputational concerns following pressured or non-consensual experiences (Gassó et al., 2020; Ringrose et al., 2013). Although consensual sexting shows weaker associations with psychological distress, it has been linked to impulsivity and risk-taking (Borgogna et al., 2023). Moreover, these associations may be bidirectional; anxiety or low self-esteem may act both as antecedents and outcomes of sexting behavior (Ayala et al., 2019; Ševčíková, 2016). Nonetheless, findings remain inconsistent, as some studies find no significant associations with self-esteem or well-being, underscoring the importance of contextual and typological distinctions in sexting involvement (Chacón-López et al., 2019; Giménez-Gualdo et al., 2022).

## Current Study

Research on sexting has largely focused on specific populations (e.g. school-aged adolescents or university students) without systematically comparing age groups, and relatively few studies adopt person-centered methodologies to identify sexting profiles or examine sexual double standards as a potential mediating mechanism. These limitations restrict understanding of how different patterns of sexting involvement, relate to well-being across age groups and gender. The present study identifies distinct profiles of sexting involvement among adolescents (ages 14–19) and emerging adults (ages 20–25), while considering gender differences within each group (Aim 1). It also examines how these profiles are associated with quality of life and mental health indicators, including depression, anxiety, and stress, with analyses conducted separately by age group and gender (Aim 2). Finally, the study examines whether endorsement of sexual double standards mediates the associations between sexting profiles and quality of life and mental health outcomes across age and gender subgroups (Aim 3).

## Methods

### Participants

The present study was based on a representative sample of 3,708 adolescents and emerging adults (51% boys, 49% girls – in the overall sample) aged between 14 and 25 years (*M* = 19.8, *SD* = 3.1). For the purposes of this study, those who had participated in at least one sexting behavior were selected (*n* = 3,534). This sample was stratified into two age groups: adolescents aged 14–19 years, representing 49.04% of the total of the sample (52.8% boys, 47.2% girls; *M* = 17.01, *SD* = 1.26), and emerging adults aged 20–25 years, comprising 50.96% (50.5% men, 49.5% women; *M* = 22.45, *SD* = 1.68).

### Procedure

Data collection for this study was funded by the Social Observatory of the “la Caixa” Foundation under the Education and Society program (LCF/PR/FS22/60010014). This study received approval from the Research Ethics Committee of the University of Seville (Reference:0359-N23).

A nationally representative sample of Spanish adolescents and emerging adults was recruited through quota-based stratified random sampling. The sampling was stratified by age group, gender, and geographical area, ensuring proportional representation based on official national demographic data. Participants were recruited online using the Computer-Assisted Web Interviewing (CAWI) technique through a national research panel with verified sociodemographic profiles of its members. The panel enabled access to respondents meeting the predefined quotas established to ensure proportional representation based on official national demographic data, according to gender, age, and autonomous communities of Spain.

In accordance with Spanish data protection legislation (Ley Orgánica 3/2018, de Protección de Datos Personales y garantía de los derechos digitales), individuals aged 14–17 years are legally permitted to provide their own consent for participation in research involving the processing of personal data. Consequently, informed consent was obtained directly from all participants, including minors, prior to accessing the questionnaire. Participation was voluntary, and respondents were informed that their answers would remain anonymous.

The survey was administrated on a secure online platform compliant with European Union (EU) data protection regulations, specifically the General Data Protection Regulation (GDPR). Data were collected anonymously from the outset, such that neither the research team nor the funding body had access to personal identifiers at any point. As compensation for their participation, respondents received a small, customary incentive, equivalent to €1.10 in market points within the survey platform, an amount intentionally modest to avoid undue influence and consistent with standard practice for minors and adults alike.

### Measures

Socio-demographic data were collected, in addition to responses to four validated measurement scales assessing sexting, quality of life, mental health, and sexual double standards:

#### Sexting 

The *Sexting Behavior and Motives Questionnaire* scale (Del Rey et al., 2021) was used to assess participants’ sexting behaviors over the past 12 months. It comprises 21 items covering four dimensions: sending, receiving, non-consensual forwarding, and victimization of non-consensual forwarding. Responses are rated on a five-point frequency scale, ranging from “1 = Never” to “5 = Daily”, with higher scores indicating greater involvement in each sexting behavior (e.g. “When I have received this type of content, I have forwarded or shared suggestive or sexual text messages from other people without consent”). The scale reliability was optimal (α_overall_ = 0.93, α_sending_ = 0.82, α_receiving_ = 0.85, α_forwarding_ = 0.89, and α_victimization_ = 0.90).

#### Quality of life 

The *KIDSCREEN-10 Index* (Ravens-Sieberer et al., 2010) was used. It comprises 10 items covering physical well-being, mood, social interactions, family and peer relationships, and school performance. Responses are rated on a five-point frequency scale, ranging from “1 = Never” to “5 = Always”, with higher scores indicating higher perceived quality of life (e.g. “Did you feel full of energy?”). The scale reliability was acceptable (α = 0.68).

#### Mental health 

The *Depression Anxiety Stress Scales* in its adapted version (Fonseca-Pedrero et al., 2010), was used to assess these three core dimensions of mental health. It comprises 21 items, each rated on a four-point Likert scale ranging from “1 = Does not apply to me at all” to “4 = Applies to me a lot most of the time”, with higher scores indicating higher levels of depression, anxiety, or stress (e.g. “I feel that I am worth very little as a person”). The scale reliability was good (α_overall_ = 0.93, α_depression_ = 0.86, α_anxiety_ = 0.83, α_stress_ = 0.79).

#### Sexual double standards 

The *Assessment of Sexual Standards Among Youth* scale (Emmerink et al., 2017), was used to assess the extent to which participants personally agree with or endorse gendered sexual norms that differentially regulate boys’/men’s and girls’/women’s sexual behavior. It comprises 19 items rated on a six-point Likert scale, ranging from “1 = Strongly Disagree” to “6 = Strongly Agree”, with higher scores reflecting stronger endorsement of sexual double standards (e.g. “Once the boy becomes sexually aroused, the girl can no longer refuse sex”). The scale reliability was adequate (α = 0.79).

### Data Analysis

All analyses were conducted using SPSS v.29, Mplus 8, and Jamovi 2.6.26. Data was first coded and cleaned in SPSS v.29, followed by descriptive analyses and internal consistency checks for all study variables.

First, sample was divided into adolescents (14–19 years) and emerging adults (20–25 years), following general age classifications used in public health research (Dick & Ferguson, 2015; World Health Organization, 2024). Then, the sub-sample of adolescents and emerging adults who had not engaged in any sexting behavior was excluded (*n*_adolescents_ = 71, 4.1%; *n*_emerging adults_ = 103, 5.7%). Girls/women were significantly more likely to be classified as not involved than boys/men in both the adolescent (2.4% vs. 1.6%) [χ² (2, *n* = 71) = 4,778, *p* = .03, V = 0.052] and emerging adult (3.5% vs. 1.9%) [χ² (2, *n* = 103) = 8,313, *p* = .004, V = 0.066] sub-samples, respectively.

Latent Profile Analyses (LPAs) were conducted in Mplus 8 to identify participant profiles within each age group (Muthén & Muthén, 2010) based on their engagement in sexting behaviors: sending, receiving, non-consensual forwarding, and victimization of non-consensual forwarding. Model fit for both the adolescent and emerging adult sub-samples was evaluated using the Akaike Information Criterion (AIC; Akaike, 1974), the Bayesian Information Criterion (BIC; Schwarz, 1978), the Lo-Mendell-Rubin adjusted likelihood ratio test (LMR-A), and entropy values. For adolescents and emerging adults, models with one to three profiles were estimated (see Table 1). The optimal number of profiles was determined based on the lowest AIC and BIC values, entropy values above 0.80 and approaching 1, and a non-significant *p*-value in the following LMR-A test, indicating no significant improvement from adding another profile (Clark & Muthen, 2009).

Gender (1 = boys/men; 2 = girls/women) differences in profile membership were analyzed in both the adolescent and emerging adult sub-samples using chi-squared test (χ²), along with Phi statistic, Cramer’s V, and adjusted standardized residuals (*p* < .05; critical value > ± 1.96) (Field, 2024).

Differences between sexting profiles were also analyzed in the variables under study among boys/men ang girls/women in both the adolescent and emerging adult sub-samples using Student’s t-test (see Table 2). Effect size was estimated using Cohen’s *d* index, categorizing it as ‘small’ between 0.2 and 0.3, ‘medium’ around 0.5 and ‘large’ from 0.8 to infinity.

Finally, to examine the mediating role of sexual double standards between sexting profile membership and indicators of quality of life and mental health (depression, anxiety, and stress), Generalized Linear Model (GLM) mediation analyses were conducted using the MedMod module in Jamovi version 2.6.26. This module, based on R, employs a path estimation approach that accounts for non-normality and estimates standard errors. For the GLM analysis, a two-phase strategy was used: first, gender was included as a covariate in each model, and, given its significant effect (see Tables 3 and 4), separate models were subsequently estimated for boys/men and girls/women (see Tables 5, 6, 7 and 8). In both phases, analyses were conducted separately by age group, using 5,000 bootstrapped 95% confidence intervals (Hayes, 2013). Mediation was significant when the interval excluded zero. All tests used a significant level of *p*-value < 0.05.

## Results

### Profiles of Sexting Behavior in Adolescents and Emerging Adults

Latent Profile Analyses (LPA) identified distinct sexting behavior subgroups among adolescents and emerging adults (see Table 1). In the adolescent and emerging adult sub-samples, both AIC and BIC values decreased progressively from the one- to the three-profile solutions. In both cases, the Lo-Mendell-Rubin adjusted likelihood ratio test (LMR-A LRT) was significant for the two-profile model but not for the three-profile model. The two-profile solution also demonstrated optimal entropy in the adolescent (0.974) and emerging adult (0.973) sub-samples, supporting its selection as the best-fitting model based on AIC, BIC, LMR-A LRT, and classification accuracy. The two identified profiles were: (1) *occasional primary sexting*, OPS (90.4%, *n* = 1,566 among adolescents; 85.1%, *n* = 1,532 among emerging adults), characterized by sporadic involvement in any form of sexting, mainly in sending one’s own content and receiving sexts directly from the sender (with low forwarding or victimization); and (2) *frequent extensive sexting*, FES (9.6%, *n* = 167 among adolescents; 14.9%, *n* = 269 among emerging adults), marked mainly by high participation in all behaviors (including sending, receiving, non-consensual forwarding and victimization) with particularly frequent receiving sexts directly from the sender, forwarding them without consent, and receiving forwarded content. Emerging adults were then more represented than adolescents in the profile *frequent extensive sexting*, with higher mean scores across all sexting behaviors.

**Table 1:** Fit indices for the LPA solutions on sexting behaviors among adolescents and emerging adults

# of profiles	AIC	BIC	LMR-A LRT (*p*)	Entropy
*Adolescents*_*N =* 1,733_				
1	17,078.748	17,133.324		
2	14,661.157	14,748.478	2,376.481 (*p* < .001)	0.974
3	12,410.145	12,530.213	2,213.542 (*p* = .17)	1.000
***Emerging adults***_***N =*1,801**_				
1	21,211.658	21,266.619		
2	17,699.523	17,757.461	3,476.832 (*p* < .001)	0.973
3	17,132.313	17,253.227	537.265 (*p* = .111)	0.780

Note. AIC = Akaike information criterion; BIC = Bayesian information criterion; LMR-A LRT = Lo–Mendell–Rubin-adjusted likelihood ratio test

#### Gender differences in sexting profile membership 

Among adolescents, more boys than girls belonged to both the OPS (*occasional primary sexting*) profile (51.3% vs. 48.7%), and the FES (*frequent extensive sexting*) profile (12.1% vs. 6.8%) [χ² (2, *n* = 1,733) = 13.854, *p* < .001, *V* = 0.089].

Among emerging adults, more women than men belonged to the OPS profile (53.4% vs. 46.6%), whereas a significantly higher proportion of men, compared to women, belonged to the FES profile (72.8% vs. 27.2%) [χ²(2, *n* = 1,801) = 63.547, *p* < .001, *V* = 0.188].

#### Gender differences in quality of life and mental health according to sexting profiles 

Descriptive results are shown in Table 2. Statistically significant differences were found for all variables studied between boys/men and girls/women and between profiles (OPS vs. FES) in both the adolescent and emerging adult sub-samples.

In terms of gender, among adolescents, girls reported lower Quality of Life than boys, but higher levels of Depression, Anxiety, and Stress. Conversely, boys scored higher than girls on Sexual Double Standards. Among emerging adults, the pattern of results was similar across women and men.

In terms of sexting profiles, adolescents in the FES profile reported lower Quality of Life than those in the OPS profile, as well as higher levels of Depression, Anxiety, Stress, and endorsement of Sexual Double Standards. Among emerging adults, the pattern between OPS and FES was comparable.

**Table 2:** Student’s t-tests of quality of life, mental health, and sexual double standards by age group (adolescents and emerging adults) and gender

		Adolescents										Emerging adults									
		Boys					Girls					Men					Women				
		*M*	*SD*	*t*	*p*	d	*M*	*SD*	*t*	*p*	d	*M*	*SD*	*t*	*p*	d	*M*	*SD*	*t*	*p*	d
**QofL**	***OPS***	3,68	,56	3,771	<0.001	0.38	3,45	,58	2,918	0.005	0.41	3,67	,55	3,548	<0.001	0.29	3,53	,56	4,246	<0.001	0.55
	***FES***	3,47	,54				3,21	,59				3,51	,59				3,22	,60			
**D**	***OPS***	1,85	,75	-7,713	<0.001	− 0.78	2,22	,81	-3,146	0.002	− 0.44	1,99	,76	-7,429	<0.001	− 0.58	2,17	,76	-5,261	<0.001	− 0.64
	***FES***	2,42	,64				2,57	,66				2,42	,63				2,65	,58			
**A**	***OPS***	1,73	,70	-7,516	<0.001	− 0.76	2,15	,75	-3,207	<0.001	− 0.44	1,79	,70	-10,567	<0.001	− 0.85	2,01	,76	-5,382	<0.001	− 0.65
	***FES***	2,26	,64				2,48	,63				2,38	,66				2,57	,56			
**S**	***OPS***	2,05	,66	-5,836	<0.001	− 0.59	2,46	,67	-2,322	0.023	− 0.27	2,18	,66	-7,053	<0.001	− 0.57	2,43	,64	-2,473	0.007	− 0.30
	***FES***	2,44	,58				2,64	,55				2,55	,57				2,62	,59			
**SDS**	***OPS***	2,22	,96	-10,928	<0.001	-1.106	1,90	,92	-5,377	<0.001	− 0.88	2,22	1,02	-13,663	<0.001	-1.10	1,91	,93	-11,921	<0.001	-1.48
	***FES***	3,30	1,05				2,75	1,15				3,33	,98				3,27	1,06			

Note. OPS = Occasional Primary Sexting; FES = Frequent Extensive Sexting; QofL = Quality of Life; D = Depression; A = Anxiety; S = Stress; SDS = Sexual Double Standards

### Impact of Sexting on Quality of Life and Mental Health, and the Role of Sexual Double Standards

#### Overall results for adolescents and emerging adults 

**Fig. 1:**
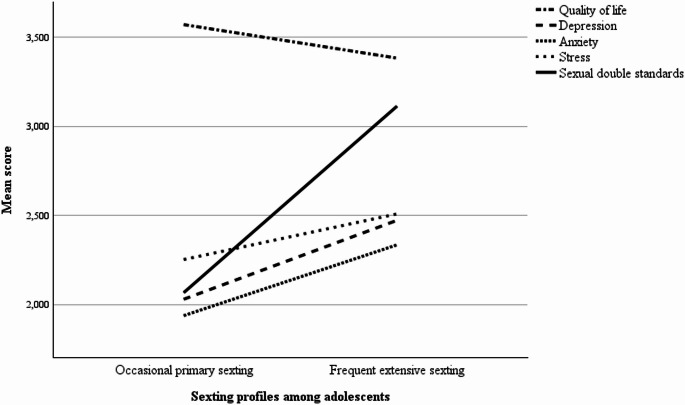
Sexting profiles among adolescents and levels of quality of life, mental health (depression, anxiety and stress), and sexual double standards

In the adolescent sub-sample (see Fig. 1 for descriptive results), the *FES profile* showed a negative direct effect on Quality of Life (*β* = -0.113, *p* < .001). In contrast, the *FES profile* showed positive direct effects on Depression (*β* = 0.173, *p* < .001), Anxiety (*β* = 0.162, *p* < .001), and Stress (*β* = 0.125, *p* < .001). Gender, entered as a covariate, demonstrated significant associations with Quality of Life (*β* = -0.197, *p* < .001) in boys, and with Depression (*β* = 0.229, *p* < .001), Anxiety (*β* = 0.277, *p* < .001), and Stress (*β* = 0.286, *p* < .001) in girls. Regarding the mediation analyses, only the *FES profile* yielded a significant indirect effect on Anxiety through endorsement of Sexual Double Standards (*β* = 0.018, SE = 0.018, 95% CI [0.009, 0.084], *p* = .012; see Table 3).

**Table 3:** Direct, indirect, component and total effects of the GLM mediation model in the adolescent sub-sample

Effect Type	Path	Estimate	SE	95% CI [LL – UL]	β	z	*p*
Outcome: _Quality of Life (QofL)_							
**Indirect**	FES → SDS → QofL	9.85e − 4	0.014	[-0.026, 0.029]	4.97e − 4	0.069	0.945
	Gender → SDS → QofL	-3.31e − 4	0.005	[-0.010, 0.009]	-2.83e − 4	-0.069	0.945
**Component**	FES → SDS	0.996	0.078	[0.837, 1.184]	0.288	12.720	< 0.001
	SDS → QofL	9.89e − 4	0.014	[-0.026, 0.029]	0.002	0.069	0.945
	Gender → SDS	-0.335	0.046	[-0.428, -0.248]	-0.164	-7.234	< 0.001
**Direct**	FES → QofL	-0.224	0.049	[-0.314, -0.135]	-0.113	-4.598	< 0.001
	Gender → QofL	-0.223	0.047	[-0.316, -0.133]	-0.113	-4.786	< 0.001
**Total**	FES → QofL	-0.223	0.047	[-0.316, -0.133]	-0.113	-4.786	< 0.001
	Gender → QofL	-0.231	0.028	[-0.282, -0.175]	-0.197	-8.377	< 0.001
**Outcome**: _**Depression (D)**_							
**Indirect**	FES → SDS → D	0.030	0.019	[-0.009, 0.069]	0.011	1.560	0.120
	Gender → SDS → D	-0.010	0.007	[-0.024, 0.003]	-0.006	-1.530	0.125
**Component**	FES → SDS	0.996	0.078	[0.838, 1.177]	0.288	12.720	< 0.001
	SDS → D	0.030	0.019	[-0.010, 0.069]	0.039	1.570	0.117
	Gender → SDS	-0.335	0.046	[-0.425, -0.254]	-0.164	-7.230	< 0.001
**Direct**	FES → D	0.467	0.066	[0.343, 0.576]	0.173	7.120	< 0.001
	Gender → D	0.366	0.038	[0.291, 0.445]	0.229	9.740	< 0.001
**Total**	FES → D	0.497	0.063	[0.381, 0.599]	0.184	7.920	< 0.001
	Gender → D	0.356	0.037	[0.279, 0.426]	0.223	9.610	< 0.001
**Effect Type**	**Path**	**Estimate**	**SE**	**95% CI [LL – UL]**	***β***	***z***	***p***
**Outcome**: _**Anxiety (A)**_							
**Indirect**	FES → SDS → A	0.046	0.018	[0.009, 0.084]	0.018	2.510	0.012
	Gender → SDS → A	-0.015	0.006	[-0.029, -0.004]	-0.010	-2.410	0.016
**Component**	FES → SDS	0.996	0.078	[0.814, 1.162]	0.288	12.720	< 0.001
	SDS → A	0.046	0.018	[0.008, 0.080]	0.062	2.560	0.011
	Gender → SDS	-0.335	0.046	[-0.427, -0.247]	-0.164	-7.230	< 0.001
**Direct**	FES → A	0.413	0.061	[0.306, 0.523]	0.162	6.760	< 0.001
	Gender → A	0.418	0.035	[0.348, 0.484]	0.277	11.930	< 0.001
**Total**	FES → A	0.458	0.058	[0.362, 0.580]	0.180	7.830	< 0.001
	Gender → A	0.402	0.035	[0.337, 0.470]	0.267	11.640	< 0.001
**Outcome**: _**Stress (S)**_							
**Indirect**	FES → SDS → S	0.021	0.017	[-0.015, 0.052]	0.009	1.270	0.203
	Gender → SDS → S	-0.007	0.006	[-0.019, 0.005]	-0.005	-1.260	0.208
**Component**	FES → SDS	0.996	0.078	[0.819, 1.169]	0.288	12.720	< 0.001
	SDS → S	0.021	0.017	[-0.015, 0.052]	0.031	1.280	0.201
	Gender → SDS	-0.335	0.046	[-0.429, -0.235]	-0.164	-7.230	< 0.001
**Direct**	FES → S	0.294	0.056	[0.195, 0.399]	0.125	5.210	< 0.001
	Gender → S	0.396	0.032	[0.337, 0.461]	0.286	12.260	< 0.001
**Total**	FES → S	0.315	0.054	[0.216, 0.407]	0.134	5.840	< 0.001
	Gender → S	0.389	0.032	[0.327, 0.454]	0.281	12.210	< 0.001

Note. FES = Frequent Extensive Sexting; SDS = Sexual Double Standards; QofL = Quality of Life; D = Depression; A = Anxiety; S = Stress

**Fig. 2:**
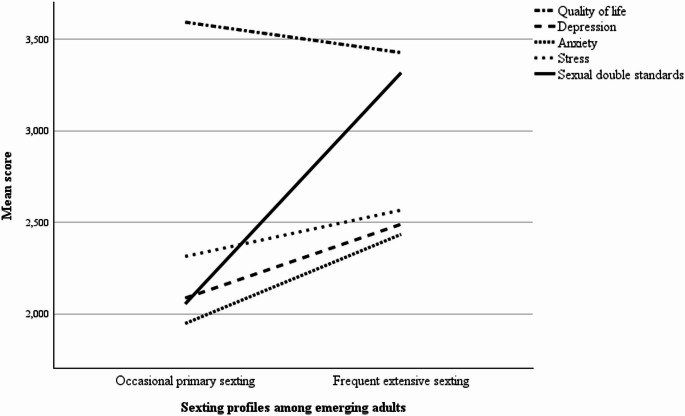
Sexting profiles among emerging adults and levels of quality of life, mental health (depression, anxiety and stress), and sexual double standards

In the emerging adult sub-sample (see Fig. 2 for descriptive results), the *FES profile* also showed a negative direct effect on Quality of Life (*β* = -0.124, *p* < .001), and positive direct effects on Depression (*β* = 0.161, *p* < .001), Anxiety (*β* = 0.212, *p* < .001), and Stress (*β* = 0.129, *p* < .001). Gender, entered as a covariate, demonstrated significant associations with Quality of Life (*β* = -0.146, *p* < .001) in men, and with Depression (*β* = 0.138, *p* < .001), Anxiety (*β* = 0.199, *p* < .001), and Stress (*β* = 0.187, *p* < .001) in women. Regarding the mediation effects, the *FES profile* yielded significant indirect effects through Sexual Double Standards on Depression (*β* = 0.051, SE = 0.021, 95% CI [0.067, 0.154], *p* < .001), Anxiety (*β* = 0.054, SE = 0.021, 95% CI [0.070, 0.156], *p* < .001), and Stress (*β* = 0.041, SE = 0.019, 95% CI [0.040, 0.116], *p* < .001; see Table 4).

**Table 4:** Direct, indirect, component and total effects of the GLM mediation model in the emerging adult sub-sample

Effect Type	Path	Estimate	SE	95% CI [LL – UL]	β	z	*p*
Outcome: _Quality of Life (QofL)_							
**Indirect**	FES → SDS → QofL	-0.012	0.016	[-0.042, 0.020]	-0.007	-0.739	0.460
	Gender → SDS → QofL	0.003	0.004	[-0.005, 0.010]	0.003	0.734	0.463
**Component**	FES → SDS	1.187	0.065	[1.050, 1.317]	0.391	18.125	< 0.001
	SDS → QofL	-0.010	0.014	[-0.036, 0.017]	-0.019	-0.739	0.460
	Gender → SDS	-0.287	0.047	[-0.374, -0.185]	-0.132	-6.126	< 0.001
**Direct**	FES → QofL	-0.197	0.041	[-0.280, -0.112]	-0.124	-4.830	< 0.001
	Gender → QofL	-0.166	0.027	[-0.215, -0.113]	-0.146	-6.103	< 0.001
**Total**	FES → QofL	-0.209	0.038	[-0.280, -0.135]	-0.131	-5.565	< 0.001
	Gender → QofL	-0.163	0.027	[-0.216, -0.110]	-0.143	-6.057	< 0.001
**Outcome**: _**Depression (D)**_							
**Indirect**	FES → SDS → D	0.110	0.022	[0.067, 0.154]	0.052	4.990	< 0.001
	Gender → SDS → D	-0.027	0.007	[-0.041, -0.015]	-0.017	-3.960	< 0.001
**Component**	FES → SDS	1.187	0.065	[1.052, 1.326]	0.391	18.130	< 0.001
	SDS → D	0.092	0.018	[0.054, 0.127]	0.132	5.190	< 0.001
	Gender → SDS	-0.287	0.047	[-0.378, -0.192]	-0.132	-6.130	< 0.001
**Direct**	FES → D	0.342	0.054	[0.242, 0.440]	0.161	6.370	< 0.001
	Gender → D	0.210	0.036	[0.130, 0.283]	0.138	5.890	< 0.001
**Total**	FES → D	0.452	0.050	[0.364, 0.535]	0.212	9.070	< 0.001
	Gender → D	0.184	0.036	[0.112, 0.251]	0.121	5.160	< 0.001
**Effect Type**	**Path**	**Estimate**	**SE**	**95% CI [LL – UL]**	***β***	***z***	***p***
**Outcome**: _**Anxiety (A)**_							
**Indirect**	FES → SDS → A	0.113	0.021	[0.069, 0.155]	0.054	5.270	< 0.001
	Gender → SDS → A	-0.027	0.007	[-0.044, -0.015]	-0.018	-4.100	< 0.001
**Component**	FES → SDS	1.187	0.065	[1.035, 1.310]	0.391	18.130	< 0.001
	SDS → A	0.095	0.017	[0.060, 0.128]	0.137	5.510	< 0.001
	Gender → SDS	-0.287	0.047	[-0.386, -0.187]	-0.132	-6.130	< 0.001
**Direct**	FES → A	0.446	0.052	[0.363, 0.549]	0.212	8.570	< 0.001
	Gender → A	0.299	0.035	[0.232, 0.368]	0.199	8.650	< 0.001
**Total**	FES → A	0.558	0.048	[0.470, 0.645]	0.266	11.570	< 0.001
	Gender → A	0.272	0.035	[0.202, 0.338]	0.181	7.870	< 0.001
**Outcome**: _**Stress (S)**_							
**Indirect**	FES → SDS → S	0.075	0.019	[0.040, 0.116]	0.041	4.000	< 0.001
	Gender → SDS → S	-0.018	0.005	[-0.032, -0.008]	-0.014	-3.410	< 0.001
**Component**	FES → SDS	1.187	0.065	[1.052, 1.307]	0.391	18.130	< 0.001
	SDS → S	0.063	0.015	[0.033, 0.096]	0.104	4.100	< 0.001
	Gender → SDS	-0.287	0.047	[-0.385, -0.197]	-0.132	-6.130	< 0.001
**Direct**	FES → S	0.238	0.047	[0.154, 0.317]	0.129	5.100	< 0.001
	Gender → S	0.246	0.031	[0.183, 0.310]	0.187	7.950	< 0.001
**Total**	FES → S	0.313	0.043	[0.236, 0.395]	0.170	7.270	< 0.001
	Gender → S	0.228	0.031	[0.164, 0.286]	0.173	7.410	< 0.001

Note. FES = Frequent Extensive Sexting; SDS = Sexual Double Standards; QofL = Quality of Life; D = Depression; A = Anxiety; S = Stress

#### Results by gender 

**Fig. 3:**
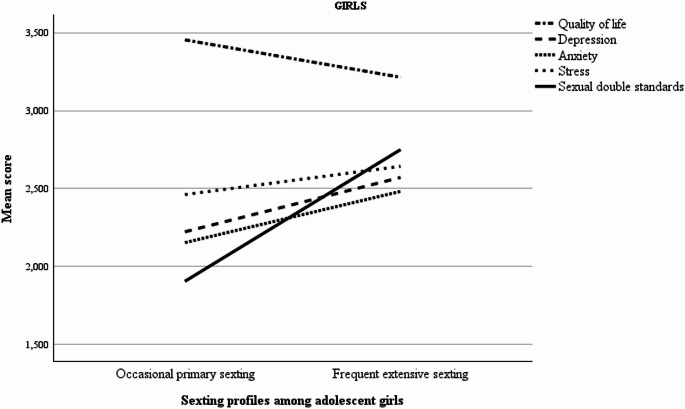
Sexting profiles among adolescent girls and levels of quality of life, mental health (depression, anxiety and stress), and sexual double standards

**Fig. 4:**
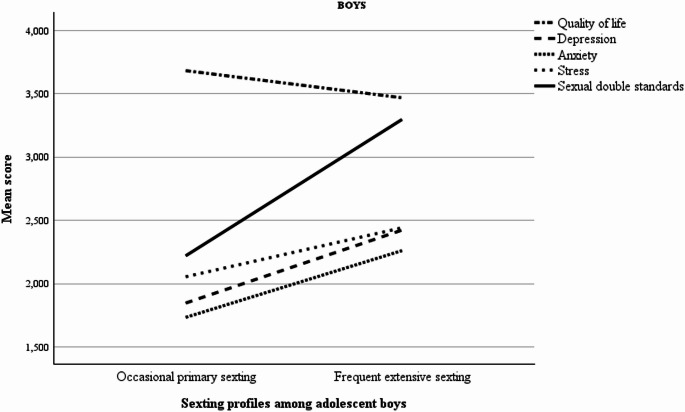
Sexting profiles among adolescent boys and levels of quality of life, mental health (depression, anxiety and stress), and sexual double standards

In the adolescent sub-sample, the FES profile was negatively associated with Quality of Life in both boys and girls (*β*_boys_ = -0.135, *p* < .001; *β*_girls_ = -0.096, *p* = .007). In terms of mental health indicators, the FES profile showed positive direct effects on Depression (*β*_boys_ = 0.237, *p* < .001; *β*_girls_ = 0.100, *p* = .005) and Anxiety (*β*_boys_ = 0.207, *p* < .001; *β*_girls_ = 0.107, *p* = .003) for both boys and girls (see Figs. 3 and 4). In contrast, the FES profile showed a positive direct effect on Stress only among boys (*β*_boys_ = 0.176, *p* < .001), whereas the effect was nonsignificant among girls (*β*_girls_ = 0.064, *p* = .072). Regarding the mediation analyses, a significant indirect effect of the FES profile through Sexual Double Standards was observed only for boys and only for Anxiety (*β*_boys_ = 0.035, SE = 0.026, 95% CI [0.019, 0.132], *p* = .004; see Tables 5 and 6).

**Table 5:** Direct, indirect, component and total effects of the GLM mediation model in the adolescent girls’ sub-sample

Effect Type	Path	Estimate	SE	95% CI [LL – UL]	β	z	*p*
Outcome: _Quality of Life (QofL)_							
**Indirect**	FES → SDS → QofL	-0.017	0.018	[-0.053, 0.019]	-0.007	-0.907	0.365
**Component**	FES → SDS	0.845	0.130	[0.589, 1.100]	0.221	6.485	< 0.001
	SDS → QofL	-0.020	0.022	[-0.062, 0.023]	-0.033	-0.916	0.360
**Direct**	FES → QofL	-0.223	0.083	[-0.385, -0.061]	-0.096	-2.705	0.007
**Total**	FES → QofL	-0.240	0.081	[-0.398, -0.082]	-0.104	-2.978	0.003
**Outcome**: _**Depression (D)**_							
**Indirect**	FES → SDS → D	0.032	0.026	[-0.013, 0.088]	0.010	1.240	0.216
**Component**	FES → SDS	0.845	0.130	[0.530, 1.149]	0.221	6.480	< 0.001
	SDS → D	0.037	0.030	[-0.018, 0.093]	0.045	1.260	0.207
**Direct**	FES → D	0.317	0.113	[0.120, 0.512]	0.100	2.800	0.005
**Total**	FES → D	0.348	0.111	[0.167, 0.516]	0.109	3.150	0.002
**Outcome**: _**Anxiety (A)**_							
**Indirect**	FES → SDS → A	0.013	0.023	[-0.032, 0.061]	0.004	0.542	0.588
**Component**	FES → SDS	0.845	0.130	[0.534, 1.164]	0.221	6.485	< 0.001
	SDS → A	0.015	0.028	[-0.039, 0.067]	0.019	0.543	0.587
**Direct**	FES → A	0.316	0.105	[0.134, 0.495]	0.107	3.011	0.003
**Total**	FES → A	0.329	0.102	[0.155, 0.506]	0.112	3.209	0.001
**Outcome**: _**Stress (S)**_							
**Indirect**	FES → SDS → S	0.011	0.021	[-0.029, 0.058]	0.004	0.516	0.606
**Component**	FES → SDS	0.845	0.130	[0.535, 1.150]	0.221	6.485	< 0.001
	SDS → S	0.013	0.025	[-0.034, 0.061]	0.019	0.517	0.605
**Direct**	FES → S	0.170	0.095	[0.006, 0.333]	0.064	1.801	0.072
**Total**	FES → S	0.181	0.092	[0.025, 0.314]	0.068	1.962	0.050

Note. FES = Frequent Extensive Sexting; SDS = Sexual Double Standards; QofL = Quality of Life; D = Depression; A = Anxiety; S = Stress

**Table 6:** Direct, indirect, component and total effects of the GLM mediation model in the adolescent boys’ sub-sample

Effect Type	Path	Estimate	SE	95% CI [LL – UL]	β	z	*p*
Outcome: _Quality of Life (QofL)_							
**Indirect**	FES → SDS → QofL	0.020	0.021	[-0.016, 0.061]	0.011	0.947	0.343
**Component**	FES → SDS	1.077	0.099	[0.887, 1.279]	0.340	10.940	< 0.001
	SDS → QofL	0.018	0.019	[-0.015, 0.054]	0.033	0.951	0.342
**Direct**	FES → QofL	-0.234	0.060	[-0.348, -0.117]	-0.135	-3.875	< 0.001
**Total**	FES → QofL	-0.214	0.057	[-0.311, -0.105]	-0.124	-3.773	< 0.001
**Outcome**: _**Depression (D)**_							
**Indirect**	FES → SDS → D	0.024	0.027	[-0.029, 0.085]	0.010	0.879	0.380
**Component**	FES → SDS	1.077	0.099	[0.888, 1.286]	0.340	10.940	< 0.001
	SDS → D	0.022	0.025	[-0.029, 0.074]	0.030	0.881	0.378
**Direct**	FES → D	0.553	0.079	[0.410, 0.682]	0.237	6.965	< 0.001
**Total**	FES → D	0.576	0.075	[0.434, 0.712]	0.247	7.718	< 0.001
**Outcome**: _**Anxiety (A)**_							
**Indirect**	FES → SDS → A	0.075	0.026	[0.019, 0.132]	0.034	2.880	0.004
**Component**	FES → SDS	1.077	0.099	[0.879, 1.295]	0.340	10.940	< 0.001
	SDS → A	0.070	0.023	[0.017, 0.112]	0.101	2.990	0.003
**Direct**	FES → A	0.452	0.074	[0.327, 0.606]	0.207	6.100	< 0.001
**Total**	FES → A	0.527	0.070	[0.412, 0.660]	0.241	7.520	< 0.001
**Outcome**: _**Stress (S)**_							
**Indirect**	FES → SDS → S	0.028	0.024	[-0.016, 0.077]	0.014	1.180	0.239
**Component**	FES → SDS	1.077	0.099	[0.882, 1.282]	0.340	10.940	< 0.001
	SDS → S	0.026	0.022	[-0.018, 0.069]	0.041	1.180	0.236
**Direct**	FES → S	0.358	0.070	[0.234, 0.493]	0.176	5.100	< 0.001
**Total**	FES → S	0.387	0.066	[0.272, 0.517]	0.190	5.840	< 0.001

Note. I = Involvement; SDS = Sexual Double Standards; QofL = Quality of Life; D = Depression; A = Anxiety; S = Stress

**Fig. 5:**
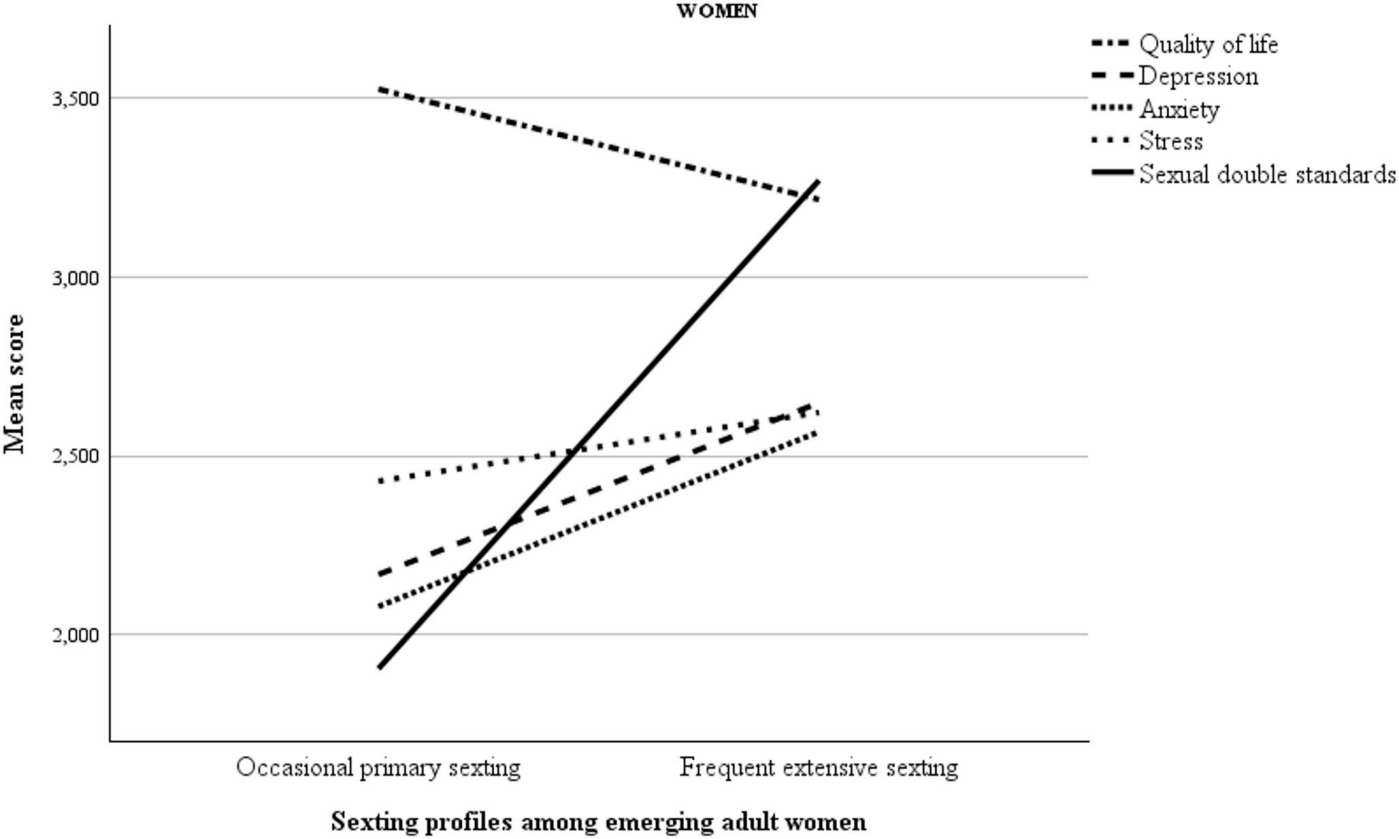
Sexting profiles among emerging adult women and levels of quality of life, mental health (depression, anxiety and stress), and sexual double standards

**Fig. 6:**
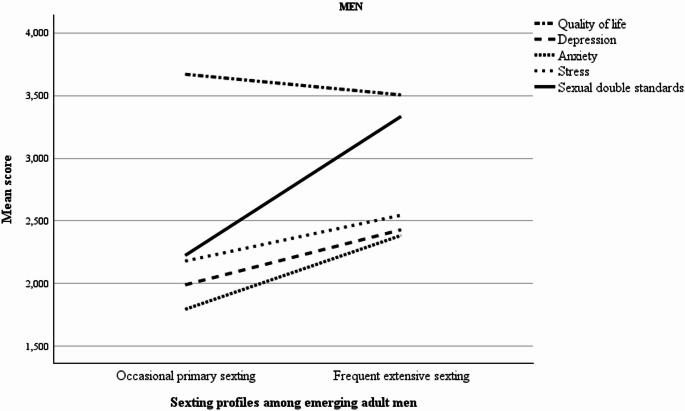
Sexting profiles among emerging adult men and levels of quality of life, mental health (depression, anxiety and stress), and sexual double standards

In the emerging adult sub-sample, the *FES profile* was negatively associated with Quality of Life for both men and women (*β*_*men*_ = -0.124, *p* < .001; *β*_*women*_ = -0.135, *p* = .007). Regarding mental health indicators, the *FES profile* showed positive direct effects on Depression (*β*_*men*_ = 0.182, *p* < .001; *β*_*women*_ = 0.130, *p* < .001) and Anxiety (*β*_*men*_ = 0.267, *p* < .001; *β*_*women*_ = 0.134, *p* < .001) in both groups (see Figs. 5 and 6). In contrast, the *FES profile* was positively associated with Stress only among men (*β*_*men*_ = 0.170, *p* < .001), whereas this effect was nonsignificant among women (*β*_*women*_ = 0.058, *p* = .104). Concerning the mediation analyses, both men and women exhibited significant indirect effects of the *FES profile* on certain mental health indicators through endorsement of Sexual Double Standards. Specifically, significant indirect effects were observed on Depression (*β*_*men*_ = 0.058, SE = 0.028, 95% CI [0.049, 0.169], *p* < .001; *β*_*women*_ = 0.044, SE = 0.038, 95% CI [0.056, 0.202], *p* = .001) and Anxiety (*β*_*men*_ = 0.064, SE = 0.026, 95% CI [0.067, 0.178], *p* < .001; *β*_*women*_ = 0.043, SE = 0.037, 95% CI [0.047, 0.209], *p* = .001). For Stress, only men showed a significant indirect effect (*β*_*men*_ = 0.058, SE = 0.024, 95% CI [0.044, 0.150], *p* < .001) through Sexual Double Standards, whereas the indirect effect among women was nonsignificant (*β*_*women*_ = 0.024, SE = 0.031, 95% CI [-0.003, 0.122], *p* = .072; see Tables 7 and 8).

**Table 7:** Direct, indirect, component and total effects of the GLM mediation model in the emerging adult women’s sub-sample

Effect Type	Path	Estimate	SE	95% CI [LL – UL]	β	z	*p*
Outcome: _Quality of Life (QofL)_							
**Indirect**	FES → SDS → QofL	-0.033	0.027	[-0.086, 0.015]	-0.016	-1.200	0.231
**Component**	FES → SDS	1.365	0.114	[1.097, 1.593]	0.371	11.930	< 0.001
	SDS → QofL	-0.024	0.020	[-0.060, 0.012]	-0.043	-1.200	0.229
**Direct**	FES → QofL	-0.276	0.073	[-0.426, -0.116]	-0.135	-3.780	< 0.001
**Total**	FES → QofL	-0.309	0.068	[-0.448, -0.163]	-0.151	-4.550	< 0.001
**Outcome**: _**Depression (D)**_							
**Indirect**	FES → SDS → D	0.121	0.038	[0.056, 0.202]	0.044	3.220	0.001
**Component**	FES → SDS	1.365	0.114	[1.083, 1.589]	0.371	11.930	< 0.001
	SDS → D	0.089	0.027	[0.043, 0.143]	0.118	3.340	< 0.001
**Direct**	FES → D	0.359	0.098	[0.194, 0.495]	0.130	3.680	< 0.001
**Total**	FES → D	0.480	0.091	[0.337, 0.633]	0.174	5.260	< 0.001
**Outcome**: _**Anxiety (A)**_							
**Indirect**	FES → SDS → A	0.119	0.037	[0.047, 0.209]	0.043	3.180	0.001
**Component**	FES → SDS	1.365	0.114	[1.088, 1.617]	0.371	11.930	< 0.001
	SDS → A	0.087	0.026	[0.033, 0.139]	0.116	3.300	< 0.001
**Direct**	FES → A	0.369	0.097	[0.203, 0.516]	0.134	3.810	< 0.001
**Total**	FES → A	0.488	0.091	[0.335, 0.624]	0.178	5.380	< 0.001
**Outcome**: _**Stress (S)**_							
**Indirect**	FES → SDS → S	0.056	0.031	[-0.003, 0.122]	0.024	1.800	0.072
**Component**	FES → SDS	1.365	0.114	[1.102, 1.624]	0.371	11.930	< 0.001
	SDS → S	0.041	0.023	[-0.002, 0.088]	0.065	1.820	0.069
**Direct**	FES → S	0.136	0.083	[-0.032, 0.274]	0.058	1.630	0.104
**Total**	FES → S	0.192	0.078	[0.050, 0.332]	0.083	2.470	0.013

Note. FES = Frequent Extensive Sexting; SDS = Sexual Double Standards; QofL = Quality of Life; D = Depression; A = Anxiety; S = Stress

**Table 8:** Direct, indirect, component and total effects of the GLM mediation model in the emerging adult men sub-sample

Effect Type	Path	Estimate	SE	95% CI [LL – UL]	β	z	*p*
Outcome: _Quality of Life (QofL)_							
**Indirect**	FES → SDS → QofL	0.004	0.020	[-0.038, 0.043]	0.003	0.204	0.838
**Component**	FES → SDS	1.109	0.081	[0.957, 1.268]	0.413	13.678	< 0.001
	SDS → QofL	0.004	0.018	[-0.034, 0.038]	0.007	0.204	0.838
**Direct**	FES → QofL	-0.169	0.049	[-0.267, -0.069]	-0.124	-3.425	< 0.001
**Total**	FES → QofL	-0.165	0.045	[-0.261, -0.080]	-0.121	-3.666	< 0.001
**Outcome**: _**Depression (D)**_							
**Indirect**	FES → SDS → D	0.106	0.028	[0.049, 0.169]	0.058	3.820	< 0.001
**Component**	FES → SDS	1.109	0.081	[0.958, 1.274]	0.413	13.680	< 0.001
	SDS → D	0.095	0.024	[0.046, 0.147]	0.139	3.980	< 0.001
**Direct**	FES → D	0.334	0.064	[0.225, 0.454]	0.182	5.190	< 0.001
**Total**	FES → D	0.439	0.059	[0.339, 0.541]	0.239	7.430	< 0.001
**Outcome**: _**Anxiety (A)**_							
**Indirect**	FES → SDS → A	0.114	0.026	[0.067, 0.177]	0.064	4.340	< 0.001
**Component**	FES → SDS	1.109	0.081	[0.959, 1.254]	0.413	13.680	< 0.001
	SDS → A	0.103	0.023	[0.059, 0.153]	0.156	4.580	< 0.001
**Direct**	FES → A	0.475	0.060	[0.368, 0.602]	0.267	7.850	< 0.001
**Total**	FES → A	0.589	0.056	[0.479, 0.687]	0.331	10.570	< 0.001
**Outcome**: _**Stress (S)**_							
**Indirect**	FES → SDS → S	0.094	0.024	[0.044, 0.150]	0.058	3.860	< 0.001
**Component**	FES → SDS	1.109	0.081	[0.942, 1.270]	0.413	13.680	< 0.001
	SDS → S	0.085	0.021	[0.042, 0.126]	0.141	4.020	< 0.001
**Direct**	FES → S	0.272	0.056	[0.170, 0.377]	0.170	4.830	< 0.001
**Total**	FES → S	0.366	0.052	[0.263, 0.455]	0.228	7.060	< 0.001

Note. FES = Frequent Extensive Sexting; SDS = Sexual Double Standards; QofL = Quality of Life; D = Depression; A = Anxiety; S = Stress

## Discussion

Sexting has become a widespread form of digital sexual expression among adolescents and emerging adults, raising important questions about its psychological, relational, and gendered implications (Chatzinikolaou & Lievens, 2021). Although research has increasingly documented the prevalence of these practices, understanding how different patterns of sexting involvement relate to well-being remains limited. In particular, relatively little attention has been given to identifying heterogeneous profiles of sexting behavior and examining how gender norms, such as sexual double standards, may shape their psychosocial consequences across developmental stages. This study addressed these gaps by identifying sexting profiles among adolescents and emerging adults and examining their associations with quality of life and mental health, while considering the mediating role of internalized sexual double standards.

### Sexting Behavior Profiles Across Adolescence and Emerging Adulthood

Across a large, nationally representative sample of Spanish youth who had engaged in at least one sexting behavior, two distinct profiles emerged consistently in both adolescents and emerging adults: an *occasional primary sexting* profile and a *frequent extensive sexting* profile. Their distribution, correlates, and gendered dynamics shed light on the developmental trajectory of sexting and its psychosocial consequences.

Among adolescents, the predominant profile was *occasional primary sexting*, consistent with research indicating that mid-teens are generally cautious about sharing erotic-sexual content (Madigan et al., 2018). The presence of an adolescent *frequent extensive sexting* profile subgroup, though proportionally smaller, is noteworthy. This subgroup displayed consistently higher involvement across all sexting practices, including non-consensual forwarding and victimization. This pattern resonates with developmental research suggesting that, in adolescence, sexting may function less as a relational or romantic practice and more as a peer-driven behavior tied to social validation, status negotiation, or entertainment, often reinforced by peer pressure and online group dynamics (Vanden Abeele, 2021). The adolescent *frequent extensive sexting* profile thus appears to represent a subset whose digital sexual expression is embedded within broader peer cultures of risk, circulation, and visibility.

In emerging adulthood, the same two-profile solution was identified, but their relative weight shifted. Although *occasional primary sexting* remained the most prevalent pattern, emerging adults showed a higher proportion of *frequent extensive sexting* compared to adolescents, indicating more diversified and frequent engagement across all sexting behaviors. This developmental shift is consistent with existing evidence showing that sexting becomes more integrated into relational, erotic, and communicative practices during emerging adulthood, a period characterized by greater autonomy, romantic exploration, and digitally mediated intimacy (Alonso & Romero, 2019). The fact that *frequent extensive sexting* is more common among emerging adults than adolescents underscore that not only does sexting become more frequent with age, but the range of practices, especially those involving forwarding and victimization, also broadens.

This profile distribution is consistent with patterns found in prior North American research, where similar relationship dynamics were observed (Galovan et al., 2018). Specifically, most individuals fell into broadly low-involvement patterns and a much smaller subgroup showed markedly higher and more diversified engagement. In this sense, although *occasional primary sexting* mainly covers forms of sexting that are relational and consensual, mostly associated with neutral or even positive psychosocial outcomes (Döring, 2014); the *frequent extensive sexting* profile reflects a high-risk subgroup whose broad engagement, particularly in coercive and secondary practices, carries significant psychological consequences. As such, this latter profile thus represents a clear target for public health interventions.

Together, these age-differentiated patterns support developmental explanations of digital sexual behaviors. The transition from predominantly cautious or situational involvement in adolescence toward more intentional, relational, and diversified engagement in emerging adulthood echoes sexual scripting, which posits that sexual behavior is shaped by evolving cultural scripts theory (Simon & Gagnon, 1986). Prior research confirms that both consensual and non-consensual sexting increase with age, likely reflecting greater autonomy, romantic exploration, and media exposure (Madigan et al., 2018). The findings refine this pattern by demonstrating not only an increase in occurrence, but also an increase in profile diversification and behavioral intensity.

### Psychological Impact of Sexting Profiles Across Developmental Stages

Beyond their descriptive interest, the profiles identified were meaningfully associated with indicators of quality of life and mental health. In both adolescents and emerging adults, membership in *frequent extensive sexting* was linked to lower quality of life and higher levels of depression, anxiety, and stress when compared with *occasional primary sexting*. Thus, a consistent pattern emerged across developmental stages, more frequent and extensive involvement in sexting, especially when it includes forwarding and victimization, was associated with worse psychological adjustment. These findings are in line with previous studies reporting that non-consensual or coercive sexting is associated with greater emotional distress, including anxiety, depression, and diminished well-being (Galanis et al., 2023; Wachs et al., 2021).

At the same time, the contrast between *occasional primary sexting* and *frequent extensive sexting* suggests that not all sexting engagement carries the same psychological cost. While the present study did not differentiate profiles by explicit motives or relational context, the pattern of results is compatible with prior work indicating that consensual sexting within trusted relationships may be relatively neutral or even positively experienced (Temple et al., 2014), whereas sexting embedded in coercive, pressured, or highly public dynamics tends to predict poorer mental health (Wachs et al., 2021). The results reinforce the notion that it is not sexting per se that is harmful, but rather the frequency, breadth, and coercive or non-consensual nature of the practices involved.

The mediation analyses further illuminate these developmental differences. Among adolescents, endorsement of sexual double standards mediated the association between *frequent extensive sexting* and anxiety, but not between *frequent extensive sexting* and depression, stress, or quality of life. This pattern could suggest that, in mid- to late adolescence, internalized gendered norms may be especially salient for anxiety responses, potentially reflecting heightened concerns about reputation, peer scrutiny, and moral judgment during this stage. In contrast, in emerging adults, the endorsement of sexual double standards mediated the associations between *frequent extensive sexting* and all three mental health indicators (depression, anxiety, and stress), pointing to a broader emotional impact. As adolescents move into emerging adulthood, gendered moral scripts around sexuality may become more deeply ingrained and intertwined with identity and relational experiences, amplifying not only anxiety but also depressive effect and perceived stress.

These findings are consistent with prior work showing that pressured or non-consensual sexting predicts psychological harm, particularly among youth who anticipate harsher social consequences and report greater emotional burden (Ayala et al., 2019). They also support the view that internalized sexual double standards exert their harmful effects most acutely when sexting occurs in coercive, non-consensual, or morally ambiguous contexts, shaping how young people interpret and emotionally process these experiences.

### Gendered Pathways: Differences Between Boys/Men and Girls/Women

The gender-stratified analyses clarify these patterns. Girls/young women consistently reported poorer quality of life and higher depression, anxiety, and stress, whereas boys/men scored higher on sexual double standards. Boys/men were also more likely to belong to the *frequent extensive sexting* profile across developmental stages. These findings corroborate previous studies indicating that boys engage in a broader repertoire of sexting behaviors, particularly those involving circulation of others’ erotic-sexual content (Frøyland et al., 2024; Klettke et al., 2019), while girls face greater emotional damage, often linked to reputational concerns and gendered judgment (Alonso Ruido, 2017).

In adolescence, belonging to the *frequent extensive sexting* profile was consistently associated with lower quality of life and higher depression and anxiety for both boys and girls, whereas its association with stress emerged only among boys. Among girls, no significant differences in stress were observed between the *frequent extensive sexting* and *occasional primary sexting* profiles, suggesting that both forms of sexting involvement may be equally stressful for them at this developmental stage. Mediation analyses further showed that sexual double standards played a role exclusively for boys, and only in relation to anxiety. A plausible explanation is that adolescent boys who internalize these norms may both legitimize certain forms of pressured or non-consensual sexting and simultaneously experience heightened anxiety when such behaviors conflict with emerging ethical concerns or fear of peer sanctions. For adolescent girls, anxiety and distress may already be strongly shaped by pressure, victimization, and reputational vulnerability (Ayala et al., 2019; Ringrose et al., 2013), leaving less explanatory room for individual differences in endorsement of sexual double standards at this developmental stage.

In emerging adulthood, *frequent extensive sexting* again predicted poorer quality of life and higher depression and anxiety for both men and women, with stress remaining significant only among men. Among women, no significant differences in stress were observed between the *frequent extensive sexting* and *occasional primary sexting* profiles, suggesting that sexting may be equally stressful for them regardless of the specific pattern of involvement. In this age group, sexual double standards mediated the associations between *frequent extensive sexting* and both depression and anxiety in men and women, while mediation for stress appeared only among men. These patterns suggest both shared and gender-specific mechanisms: internalized sexual double standards may amplify internalizing symptoms across genders when sexting becomes extensive, but they seem particularly relevant for men’s stress responses. This may reflect the persistence of gendered scripts that normalize male sexual assertiveness while simultaneously generating tension when such behaviors lead to relational conflict, moral dissonance, or potential social or legal repercussions.

These findings highlight the value of person-centered approaches in sexting research. Profiling clarifies which forms of sexting are most harmful, to whom, and under what circumstances. Such insights should inform prevention efforts addressing both sexting risks and the gendered dynamics that shape them.

### Implications

This research underscores the need for prevention and intervention strategies that address not only sexting behaviors but also the gendered norms that shape them. Universal school programs should focus on digital consent and relational ethics, while more selective efforts may target adolescents who show broader or non-consensual involvement. In tertiary education, relationship-focused modules can help distinguish consensual and intimate exchanges from retaliatory practices. Finally, public policies should prioritize responses to high-risk patterns such as extensive sexting involvement, strengthening protections against non-consensual forwarding and supporting gender-sensitive digital citizenship.

### Limitations

Several limitations must be acknowledged. First, the study relied on a cross-sectional design, which limits causal inference. Second, all data were obtained through self-report measures, which may be susceptible to biases such as social desirability or recall inaccuracies. Third, although the overall sample was nationally representative, the analytic subsamples may still limit generalizability to other cultural contexts. In addition, this study did not assess the relational context of sexting (e.g. whether exchanges occurred with romantic partners, peers, or acquaintances), which may shape both the meaning and psychological impact of sexting behaviors. Future research would benefit from incorporating this dimension to refine the interpretation of sexting profiles. Furthermore, the internal consistency of the quality of life scale was relatively low in this sample, which is probably related to its multidomain composition, so the interpretation of results needs to be taken with caution.

## Conclusion

Sexting is a common form of digital sexual expression during adolescence and emerging adulthood, yet prior research has rarely captured the heterogeneous patterns of sexting involvement or examined how sexual double standards shape their associations with well-being across developmental stages. This study addressed this gap by applying a person-centered approach to identify profiles of sexting involvement and to examine their links with quality of life, mental health, and sexual double standards, while considering gender differences. Two sexting profiles were identified across adolescents and emerging adults: a pattern characterized by occasional, primarily consensual exchanges and another marked by frequent and extensive engagement, including non-consensual forwarding and victimization. These profiles showed markedly different psychosocial correlates, with frequent and extensive involvement consistently associated with lower quality of life and higher levels of depression, anxiety, and stress. Sexual double standards emerged as a key mechanism linking sexting involvement with mental health outcomes, particularly among males, while girls and young women reported poorer mental health across profiles. Taken together, these findings show that sexting is a heterogeneous practice whose implications for the well-being of adolescents and emerging adults depend on patterns of involvement, developmental stage, and gender norms.

## Data Availability

The datasets generated and/or analyzed during the current study are not publicly available but are available from the corresponding author on reasonable request.
